# Plasma Metabolite Profiles in First Episode Psychosis: Exploring Symptoms Heterogeneity/Severity in Schizophrenia and Bipolar Disorder Cohorts

**DOI:** 10.3389/fpsyt.2020.00496

**Published:** 2020-06-05

**Authors:** Helena P. G. Joaquim, Alana C. Costa, Leda L. Talib, Frederik Dethloff, Mauricio H. Serpa, Marcus V. Zanetti, Martinus van de Bilt, Christoph W. Turck

**Affiliations:** ^1^ Laboratory of Neuroscience LIM-27, Department and Institute of Psychiatry, University of Sao Paulo Medical School, Sao Paulo, Brazil; ^2^ Instituto Nacional de Biomarcadores em Neuropsiquiatria (INBioN), Conselho Nacional de Desenvolvimento Científico e Tecnológico, Sao Paulo, Brazil; ^3^ Department of Translational Research in Psychiatry, Max Planck Institute of Psychiatry, Munich, Germany; ^4^ Laboratory of Psychiatric Neuroimaging LIM-21, Department and Institute of Psychiatry, University of Sao Paulo Medical School, Sao Paulo, Brazil; ^5^ Hospital Sírio-Libanês, São Paulo, Brazil

**Keywords:** metabolomics, biomarkers, psychosis, schizophrenia, bipolar disorder

## Abstract

**Introduction:**

The first symptoms of psychosis are frequently shared amongst several neuropsychiatry disorders, which makes the differentiation by clinical diagnosis challenging. Early recognition of symptoms is important in the management of psychosis. Therefore, the implementation of molecular biomarkers will be crucial for transforming the currently used diagnostic and therapeutic approach, improving insights into the underlying biological processes and clinical management.

**Objectives:**

To define a set of metabolites that supports diagnosis or prognosis of schizophrenia (SCZ) and bipolar disorder (BD) at first onset psychosis.

**Methods:**

Plasma samples from 55 drug-naïve patients, 28 SCZ and 27 BD, and 42 healthy controls (HC). All participants underwent a seminaturalistic treatment regimen, clinically evaluated on a weekly basis until achieving clinical remission. All clinical or sociodemographic aspects considered for this study were equivalent between the groups at first-onset psychosis time point. The plasma samples were analyzed by untargeted liquid chromatography-tandem mass spectrometry (LC-MS/MS) using reversed-phase and hydrophilic interaction chromatography. The acquired molecular features were analyzed with MetaboAnalyst.

**Results:**

We identified two patient groups with different metabolite profiles. Both groups are composed of SCZ and BD patients. We found differences between these two groups regarding general symptoms of PANSS score after remission (p = 0.008), and the improvement of general symptoms (delta of the score at remission minus the baseline) (−0.50 vs. −0.33, p = 0.019).

**Conclusion:**

Our results suggest that plasma metabolite profiles cluster clinical remission phenotypes based on PANSS general psychopathology scores.

## Introduction

The molecular pathogenesis of psychosis is not fully understood. First symptoms are common amongst neuropsychiatric disorders which makes clinical diagnosis challenging (1).

Naturalistic follow-up studies have found highly divergent outcomes in first-episode psychosis (FEP) (2–4). Schizophrenia (SCZ) and bipolar disorder (BD) share genetic contributions (5) and clinical symptoms, including psychosis and affective dysregulation (6), and cognitive impairment (7–9). The challenge for the clinician is to distinguish different FEP in order to make the best choices for individual patients. This requires sensitive and specific biomarkers. In fact, a combination of multiple biomarkers may better reflect etiology and provide improved insights into the underlying biological processes (10, 11).

Several recent studies have used metabolomics in an attempt to identify biomarkers for SCZ and BD (12–15). Increased serum (16–18) and plasma (19–21) fatty acid levels have been reported in patients with SCZ compared to healthy controls (HC). Reduced phosphatidylcholine (19, 22, 23) and phosphatidylethanolamine levels (24) were found in SCZ patients. However, Cai et al. (19) reported increased lysophosphatidylcholine levels in patients with SCZ. Recently, in a targeted study, our group reported that plasma phosphatidylcholine (PC aa C26:0, PC aa C38:4, PC aa C34:3) and acylcarnitine (C16-OH) levels may be useful for differentiating SCZ and BD patients (13).

In the current study, we have compared plasma metabolite profiles from SCZ and BD patients and HC to define a set of metabolites that may aid in their differentiation. The untargeted search for metabolites can provide us with a broader and more comprehensive view of the metabolome related to the psychiatric disorders studied and complement the results previously obtained.

## Materials and Methods

### Subjects

The open-label-study was conducted at the Institute of Psychiatry, University of Sao Paulo Medical School, Brazil. The sample comprised of 55 drug-naïve patients (28 SCZ and 27 BD) and 42 healthy controls. All participants were <60 years old and middle-income, community-dwelling subjects from the hospital neighborhood area. All subjects provided written informed consent prior to inclusion in the study. The study was approved by the Local Ethics Committee of the University of Sao Paulo (CAPPesq N° 943.883) and performed in accordance with the Helsinki declaration. The SCZ diagnosis was established according to the Diagnostic and Statistical Manual of Mental Disorders (DSM-IV) (25) and SCID-I/P-Structured Clinical Interview Disorders Axis I for DSM–IV 2.0 was used to confirm the diagnosis (26). Psychopathology was assessed using the Positive and Negative Symptoms Scale (PANSS) (27), including the positive and negative subscales and general psychopathology. The Hamilton Depression Rating Scale (HAM-D) (28) and Young's Mania Rating Scale (YMRS) (29) assessed depressive and manic symptoms for BD patients. Subjects with other psychiatric or neurological disorders were excluded.

Subsequently, all patients underwent a seminaturalistic antipsychotic treatment based on the recommendations of the International Psychopharmacology Algorithm Project (IPAP; www.ipap.org). Treatment regimen was strictly defined and the dosage was determined by the clinicians as in their usual practice. For the first trial, either haloperidol or risperidone were chosen. Following the IPAP SCZ algorithm, whenever no clinical remission was achieved after 4 to 6 weeks using the highest dosage tolerated by the patient according to clinician judgment, the first medication was changed to olanzapine.

FEP subjects were then clinically evaluated weekly by a psychiatrist using the PANSS until achieving clinical remission. The remission criteria used were proposed by Andreasen et al. (30), with the exception of the length of time. The subjects should present no more than mild symptom severity (PANSS score <4) in the core items: delusions, mannerisms/posturing, conceptual disorganization, hallucinatory behavior, social withdrawal, lack of spontaneity, blunted affect, and unusual thought content. Once the remission criteria were fulfilled, FEP subjects were evaluated every week until completing a month of remission.

The sociodemographic characteristics of the patients and controls are summarized in **Table 1**. All outcome variables were controlled for years of education and gender.

**Table 1:** Socio-demographic characteristics of patients and controls.

	SCZ (n=28)	BD (n=27)	HC (n=42)	*p*
Gender (M/F)	17/11	5/22	24/18	**0.002**
Age (mean ± sd)	26.0 ± 7.4	28.9 ± 5.6	27.7 ± 5.9	0.225
Education (mean ± sd)	10.8 ± 3.5	13.7 ± 2.1	13.1 ± 3.1	**0.001**
PANSS (mean ± sd)	78 ± 22	–	–	–
PANSS - Positive symptoms (mean ± sd)	19 ± 5	–	–	–
PANSS - Negative symptoms (mean ± sd)	18 ± 8	–	–	–
HAM-D (mean ± sd)	–	15 ± 8	–	–
YMRS (mean ± sd)	–	9 ± 8	–	–

SCZ, schizophrenia; BD, bipolar disorder; HC, healthy controls; M, male; F, female; PANSS, Positive and Negative Syndrome Scale; HAM-D, Hamilton Depression Rating Scale; YMRS, Young Mania Rating Scale; sd, standard deviation. Bold font indicates significant p-value.

### Sample Collection and Preparation

Blood samples were collected in EDTA-coated tubes (Vacuntainer, Becton Dickinson; Franklin Lakes, NJ, USA) after 8-h fasting. Samples were centrifuged at 20°C and 1,800 *g* for 15 min. One hundred microliter of blood plasma samples were incubated with 400-µl cold methanol for 2 h at 4°C and centrifuged at 16,000 *g* for 10 min at 4°C. The supernatant was transferred to a new tube and dried by vacuum concentration. The samples were stored at −80°C until further processing.

### Metabolomics Analysis

Reversed phase analysis: Sample extracts were reconstituted in 100 µl of methanol and centrifuged for 4 min at 10,000 rpm and analyzed using an Agilent Technologies 1100 HPLC system (Agilent Technologies; Santa Clara, CA, USA) coupled to a Bruker Impact II TOF MS system (Bruker Corporation; Billerica, MA, USA) controlled by Bruker Hystar 3.2 Software. Eluent A was H_2_O with 0.1% formic acid and eluent B acetonitrile with 0.1% formic acid. The gradient was run with a flow rate of 500 µl*min^-1^ over a Phenomenex Kinetex C18 3 × 100 mm column, 2.6-µm particle size, 100 Å pore size (Phenomenex Inc., Torrance, CA, USA), and a Phenomenex Kinetex C18 guard column, both heated to 40°C in the LC oven. Samples were kept at 6°C in the sample rack until injection of 5 µl into the injection loop of the HPLC. Samples were eluted isocratically for 2 min with 5% of eluent B followed by a 25-min gradient to 95%, 95% for 5 min followed by 6-min equilibration at 5% of eluent B.

### Mass Spectrometry

Samples were introduced splitless into the ESI source. Compounds were ionized with an end plate offset of −500 V, a capillary voltage of 4,500 V in positive mode. The nebulizer gas N_2_ flow was 11 Lmin-1, 3.5 bar pressure heated to 220°C. Bruker Compass 1.9 acquired the profile data with a spectra rate of 1 Hz (full scan) and a mass range from 30 to 1,300 *m/z*. MS/MS data were generated of the five most intense ions selected for fragmentation within a mass range of ±1–3 Da, 20–25 eV collision energy and 5-Hz spectra rate. The mass accuracy was adjusted by internal calibration using sodium format clusters in ESI+ and ESI- modes with Bruker DataAnalysis 4.4 software after calibration with Proteowizard (http://proteowizard.sourceforge.net).

For detect instrumental variations, a quality control (QC) samples was analyzed, consisting of a pool of all samples (SCZ, BD, and HC), prepared by mixing 5 µl of each sample. QC sample preparation was performed equally as for the other samples. The QC samples were injected three times at the beginning of the batch analyses, at every five injections, and once at the end of the batch.

### Data Processing and Statistical Analyses

Comparison of patients' and controls' socio-demographic characteristics was performed with Chi-square test for categorical variables and ANOVA for quantitative variables using SPSS (Statistical Package for Social Sciences, for Windows, v. 14, Chicago, IL). Significance level was p < 0.05.

For data preprocessing we used MZmine 2 (31) with the Automated Data Analysis Pipeline (ADAP) deconvolution algorithm and the CAMERA (32) pseudo-spectra annotation. Only features with at least two isotopes were kept for further analysis. Aligned data was exported as .csv file for further processing and normalized to the average sample intensity. To track instrument performance we used QC samples that were manually evaluated.

The data including retention time, the *m/z* ratio and the peak intensities of each feature in each sample was loaded into Metaboanalyst (https://www.metaboanalyst.ca). For the analysis, the intensity values ​​were normalized by sum and Pareto scale (33). Multivariate exploratory analyzes were performed using Principal Components Analysis (PCA) and Partial Least Square Discriminant Analysis (PLS-DA). The PCA is applied to check outliers, trend of clusters and even choice of the most relevant set of variables for the construction of the model. Specifically, PLS-DA is often used in metabolomics studies because it provides discriminatory characterization considering all variables in two or more supervised groups (34). The differential features obtained from PLS-DA plot were further evaluated by Variable Importance in Projection (VIP) and Student's t-test (95% of confidence) followed by signal-to-noise ratio (S/N) verification. Variables with VIP score ≥ 1 and p-value ≤ 0.005 for the t-test were considered for the classification. From the analysis with PCA we observed that the existing separation corresponded to the intensity of the severity of the symptoms. Therefore, we use this label to obtain the PLS-DA.

## Results

Using an untargeted metabolomics approach, we were able to detect 2,444 metabolite features and 607 (0.5%) missing values. In order to track any instrument variation, we ran several blank and QC samples as part of the sequence. QC samples did not show any retention time shifts and only mild variations in intensity. Only features with intensities that were twice as high as the blank mean value in at least 75% of all samples were kept. This resulted in the removal of 31 features.

No significant metabolite profiles that differentiate patients with SCZ, BD and HC were identified (Q^2^=−0.153 and R^2^=0.943) (**Supplementary Figures 1A, B**). Removing the control group also did not result in stratification of the SCZ and BD patient groups (Q^2^ = 0.251 and R^2^ = 0.915) (**Supplementary Figures 2A, B**).

**Figure 1:**
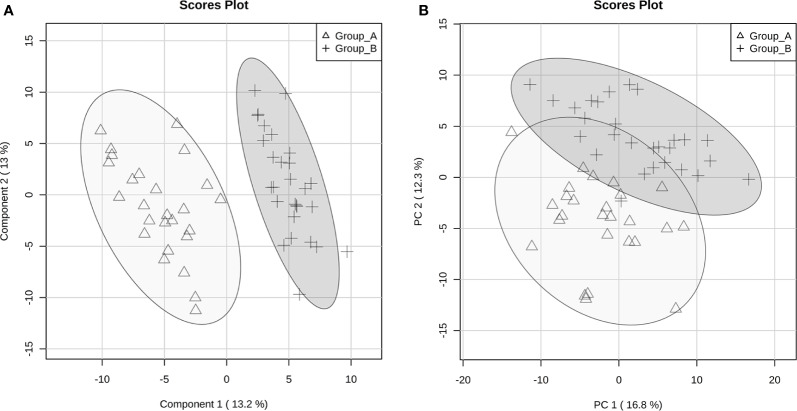
Principal Components Analysis (PCA) **(A)** and Partial Least Square Discriminant Analysis (PLS-DA) **(B)** score plots of discriminant metabolites between groups A and B according to the severity of clinical symptoms.

However, we observed different group stratification, with both Groups A and B composed of SCZ and BD patients and no differences with regard to socio-demographic and clinical data (**Figures 1A, B**). The designation of groups A and B was arbitrary and based on metabolite profile only. Interestingly, these two groups showed differences in PANSS general score (p=0.008) and also delta (remission *minus* baseline) (−0.50 vs. −0.33, p=0.019) after symptom remission (**Table 2**).

**Table 2:** Socio-demographic characteristics and clinical assessment of patients and controls according to the severity of clinical symptoms.

	Group A (n=26)	Group B (n=28)	*p*
Gender (M/F)	9/17	12/16	0.586
Age (mean ± sd)	26.8 ± 6.2	28.3 ± 7.1	0.432
Education (mean ± sd)	12.6 ± 2.9	11.9 ± 3.5	0.396
Duration of untreated psychosis in days (mean ± sd)	32.0 ± 35.5	26.7 ± 56.6	0.257
Age of onset (mean ± sd)	22.7 ± 7.4	21.6 ± 6.4	0.706
Diagnostic (schizophrenia/bipolar disorder)	13/13	14/14	1.000
Bipolar subtype distribution	7/6	6/8	0.705
Remission time in days (mean ± sd)	88 ± 64	70 ± 57	0.410
Illness duration in days (mean ± sd)	53.3 ± 46.9	82.5 ± 105.6	0.394
*Baseline scales* (mean ± sd)			
PANSS	80.0 ± 20.6	70.9 ± 19.1	0.246
PANSS – Positive symptoms	20.2 ± 5.5	18.6 ± 4.8	0.409
PANSS – Negative symptoms	17.8 ± 7.8	16.2 ± 6.8	0.586
PANSS – General symptoms	42.0 ± 11.0	40.1 ± 11.1	0.660
HAM-D	17.6 ± 6.9	13.0 ± 9.1	0.158
YMRS	7.1 ± 7.5	10.9 ± 9.3	0.251
*After clinical remission* (mean ± sd)			
PANSS	38.9 ± 7.9	45.5 ± 7.9	0.052
PANSS – Positive symptoms	8.4 ± 1.4	9.4 ± 1.8	0.191
PANSS – Negative symptoms	10.8 ± 4.7	11.3 ± 4.85	0.819
PANSS – General symptoms	19.7 ± 3.4	24.6 ± 4.0	**0.008**
HAM-D	3.1 ± 2.5	3.5 ± 3.0	0.738
YMRS	0.6 ± 1.0	2.0 ± 3.0	0.182
Δ *Clinical Scores* (mean ± sd)			
PANSS	−0.47 ± 0.14	−0.35 ± 0.15	0.079
PANSS – Positive symptoms	−0.51 ± 0.22	−0.49 ± 0.17	0.803
PANSS – Negative symptoms	−0.31 ± 0.33	−0.16 ± 0.44	0.400
PANSS – General symptoms	−0.51 ± 0.11	−0.33 ± 0.18	**0.019**
HAM-D	−0.63 ± 0.63	−0.78 ± 0.19	0.502
YMRS	−0.87 ± 0.32	−0.78 ± 0.38	0.628

M, male; F, female; sd, standard deviation; PANSS, Positive and Negative Syndrome Scale; HAM-D, Hamilton Depression Rating Scale; YMRS, Young Mania Rating Scale. Bold font indicates significant p-value.

**Figure 2:**
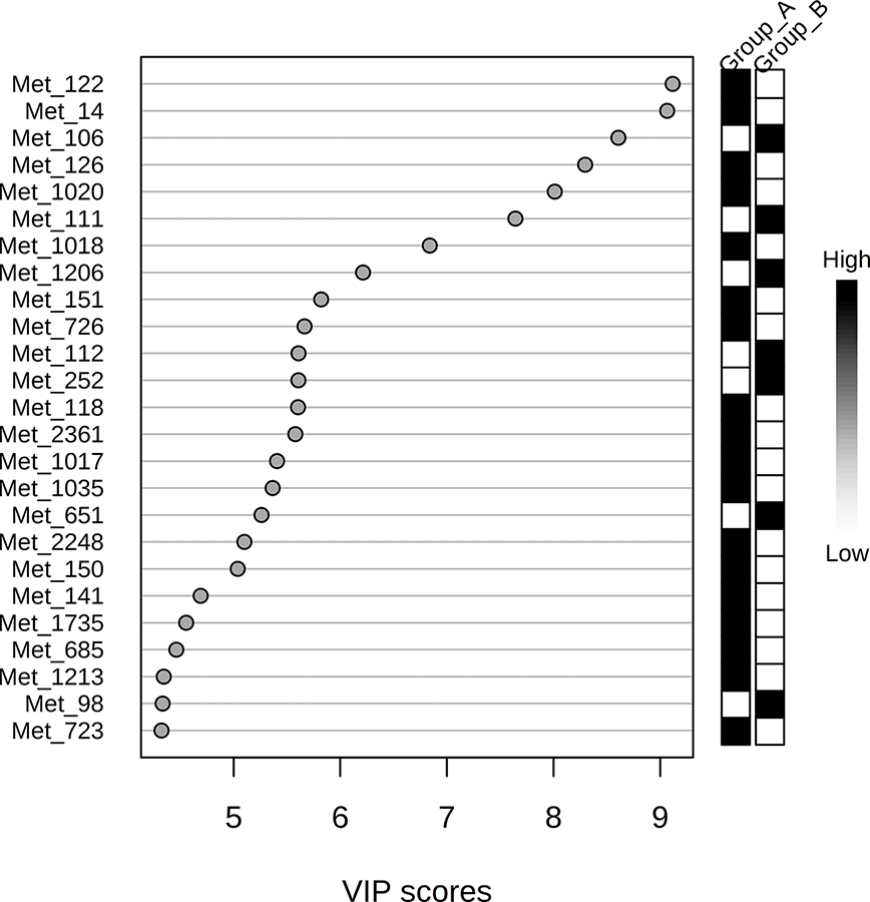
Variable Importance in Projection (VIP) score visualization for 25 differential features for groups A and B.

The 220 statistically differential features between the groups (and with no significant differences between experimental and theoretical masses) were determined and classified by multivariate analysis. PCA and PLS-DA were built excluding QC samples from data treatment. The variation in the PCA scores plot was 0.168 for PC1 and 0.123 for PC2. The PLS-DA model was validated with 86.7% predictability (Q^2^ = 0.867 and R^2^ = 0.973). After determination of differential features by PLS-DA based on group separation, we loaded VIP scores analysis (**Figure 2**), Student's t-test and S/N (signal/noise) verification. We were able to verify that 255 features were upregulated in Group A, while only 14 features presented increased levels in Group B considering a fold-change threshold ≥ 2.

## Discussion

Given the complexity and heterogeneity of psychiatric disorders, a combination of multiple biomarkers may better reflect etiology and improve insights into the underlying biological processes (10, 11). Here we used LC-MS-based metabolite profiling to gain a deeper understanding of global variation in psychosis, and to detect potential markers that facilitate its diagnosis and/or outcome. We were unable to identify outcome biomarkers in first episode psychosis. However, we found two diagnostic-mixed groups based on the acquired metabolite profiles. All clinical and socio-demographic aspects considered for this study were equivalent between the groups at the first episode time point. Interestingly, after clinical remission, we found greater improvement in General Psychopathology PANSS scores in group A.

The General Psychopathology Scale complements the positive-negative assessment in PANSS as it is a set of nonspecific symptoms representing the severity of distinct positive and negative manifestations. It provides a separate but parallel measure of disease severity, serving as a control measure for interpreting syndromic scores (27). It is a parameter that considers positive manifestations (i.e., anger and increased noncommunicative movements) and negative manifestations (for example, dull facial expression, poor eye contact, and lack of emotional relationship) in a collective and complementary way. The score on this subscale has already been positively correlated with psychosis and other serious disorders in patients' first-degree relatives. Several studies have proposed that some of the 30 items are the most predictive. For instance Lefort-Besnard et al. (35) showed a subset of eleven items as the most predictive for disease severity based, of which nine were part of the General Psychopathology Scale: emotional withdrawal, anxiety, guilt feelings, unusual thought content, lack of judgement and insight, and disturbance of volition. Therefore, the metabolite profile depicted in Group A might represent a predictor of better outcome, independently of the diagnosis.

The cluster-based analysis has been widely used to identify patient subgroups that explain clinical differences (36). Clusters based on neurocognition have been extensively explored (8, 37–40). It seems consistent that SCZ and BD patients tend to show similar patterns of neuropsychological impairments compared to controls (41, 42). Although the symptoms are more evident in SCZ than in BD, studies suggest that these effects lack diagnostic specificity (8, 39, 43–45). Recently, Chan et al. (46) performed a cluster analysis based on premorbid adjustment trajectories. All clusters had SCZ and BD, corroborating that clinical differentiation is not specific.

Our data reveal a different metabolite profile of patients who will progress to more severe psychotic symptoms which can already be seen in plasma before the clinical outcome, and thereby can assist in treatment. Unfortunately we were unable to identify the relevant metabolites with unambiguous certainty. Using exploratory approaches, we performed a putative identification of some metabolites and found that several of them are part of the same class of metabolites already described by our group as altered in psychoses including carnitines and phosphatidylcholines (13).

The fact that distinct clinical phenotypes are due to biological differences legitimates the search for biomarkers (47, 48). Since metabolite levels are sensitive to subtle perturbations during disease, metabolomics provides a powerful approach to assess these changes on the molecular level (49). The metabolomics approach has been documented in several reports on biomarkers for psychiatric disorders, including BD, depression, and SCZ (50–52).

A recent systematic literature review of SCZ metabolite biomarkers pointed to at least 63 studies using metabolomics (14). A systematic review performed by Li et al. (53) identified 10 molecules as potential biomarkers of psychosis. Our group has reported a set of 4 metabolites that differentiate SCZ and BD patients in first onset psychosis (13). Wang et al. (12) found a set of six metabolites that differentiate SCZ patients from HC. Compared to SCZ, mood disorders have been less investigated using metabolomics. A meta-analysis involving data from eight studies identified a 20-biomarker panel that differentiates BD from SCZ and depression patients (54). Our results reveal a metabolite profile in the very first psychosis episode that may assist general psychopathology prognosis and monitoring treatment response.

The main limitations of our study are the small sample size for the diagnostic groups and the missing clinical scales for some subjects. Therefore, the results need to be replicated in an independent and larger sample cohort. The basic biochemical data (complete blood count, liver enzymes, serum vitamin B12, HIV serology, and kidney and thyroid function) was considered as inclusion or exclusion criteria, but not correlated with the metabolomics findings.

## Data Availability Statement

The datasets generated for this study are available on request to the corresponding authors.

## Ethics Statement

The studies involving human participants were reviewed and approved by Local Ethics Committee of the University of Sao Paulo. The patients/participants provided their written informed consent to participate in this study.

## Author Contributions 

CT and HJ designed the study. HJ, AC, and LT performed literature searches, wrote the first draft of the manuscript, and undertook the statistical analysis. FD performed the metabolomics analyses. MS, MZ, and MB were responsible for the clinical assessment. All authors contributed to and approved the final manuscript.

## Funding 

This study was supported by the Fundação de Amparo à Pesquisa do Estado de São Paulo - FAPESP (Grants No. 2014/20913-3 and 2013/103509), Instituto Nacional de Biomarcadores em Neuropsiquiatria (INBioN - Grant No. 2014/50873-3) and the Max Planck Society. The Laboratory of Neuroscience receives financial support from Associação Beneficente Alzira Denise Hertzog da Silva (ABADHS).

## Conflict of Interest

The authors declare that the research was conducted in the absence of any commercial or financial relationships that could be construed as a potential conflict of interest.

## Supplementary Material

Supplementary Figure 1 PCA **(A)** and PLS-DA **(B)** scores plot of SCZ (gray), BD (light gray) and HC (white) samples.

Supplementary Figure 2 PCA **(A)** and PLS-DA **(B)** scores plot of SCZ (gray) and BD (light gray) samples.
