# Supplementary figures and images for: Plasma Metabolite Profiles in First Episode Psychosis: Exploring Symptoms Heterogeneity/Severity in Schizophrenia and Bipolar Disorder Cohorts
Source: Front Psychiatry. 2020 Jun 5;11:496. doi: 10.3389/fpsyt.2020.00496 (PMC7290160; doi:10.3389/fpsyt.2020.00496)

1A)

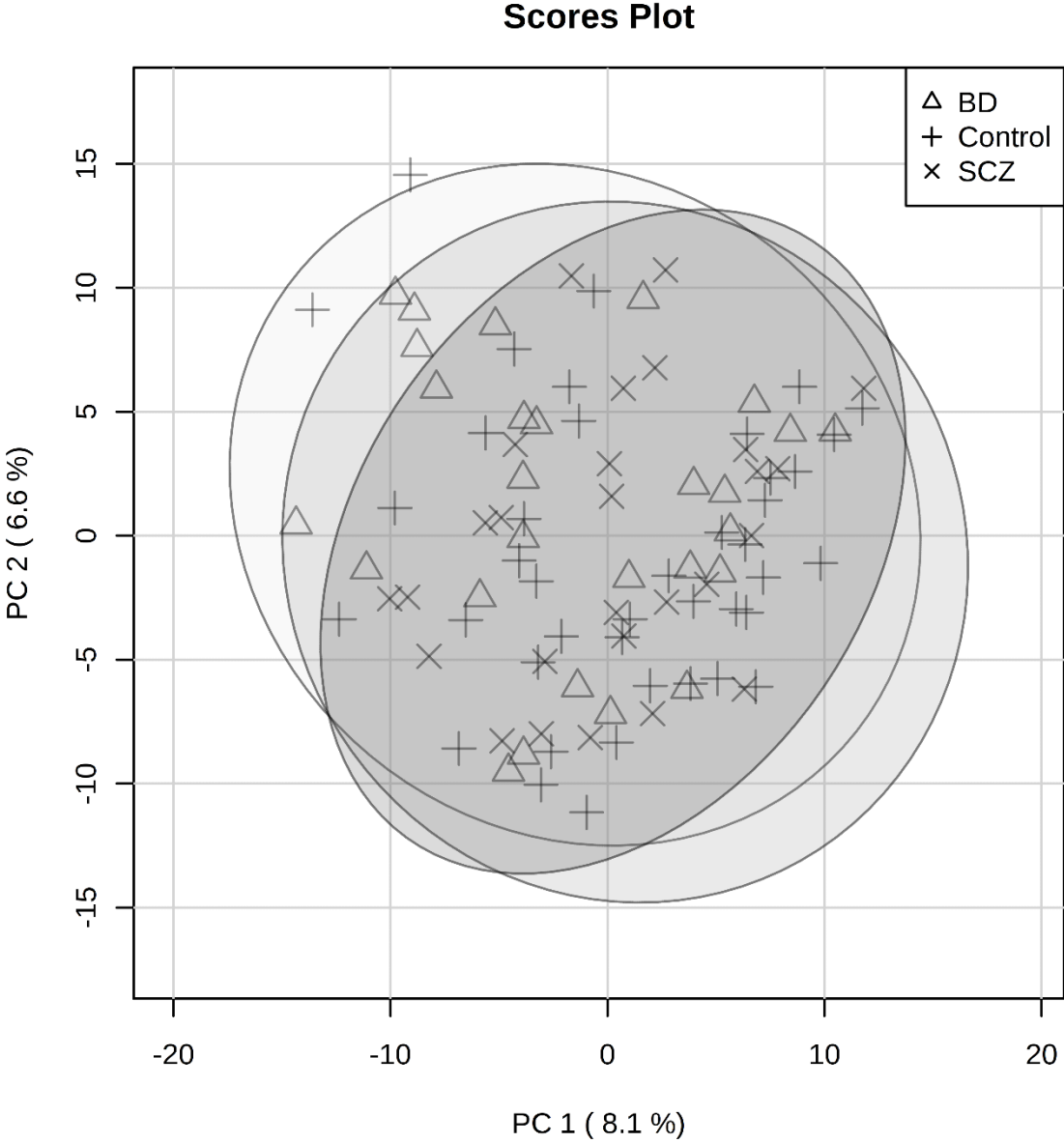

1B)

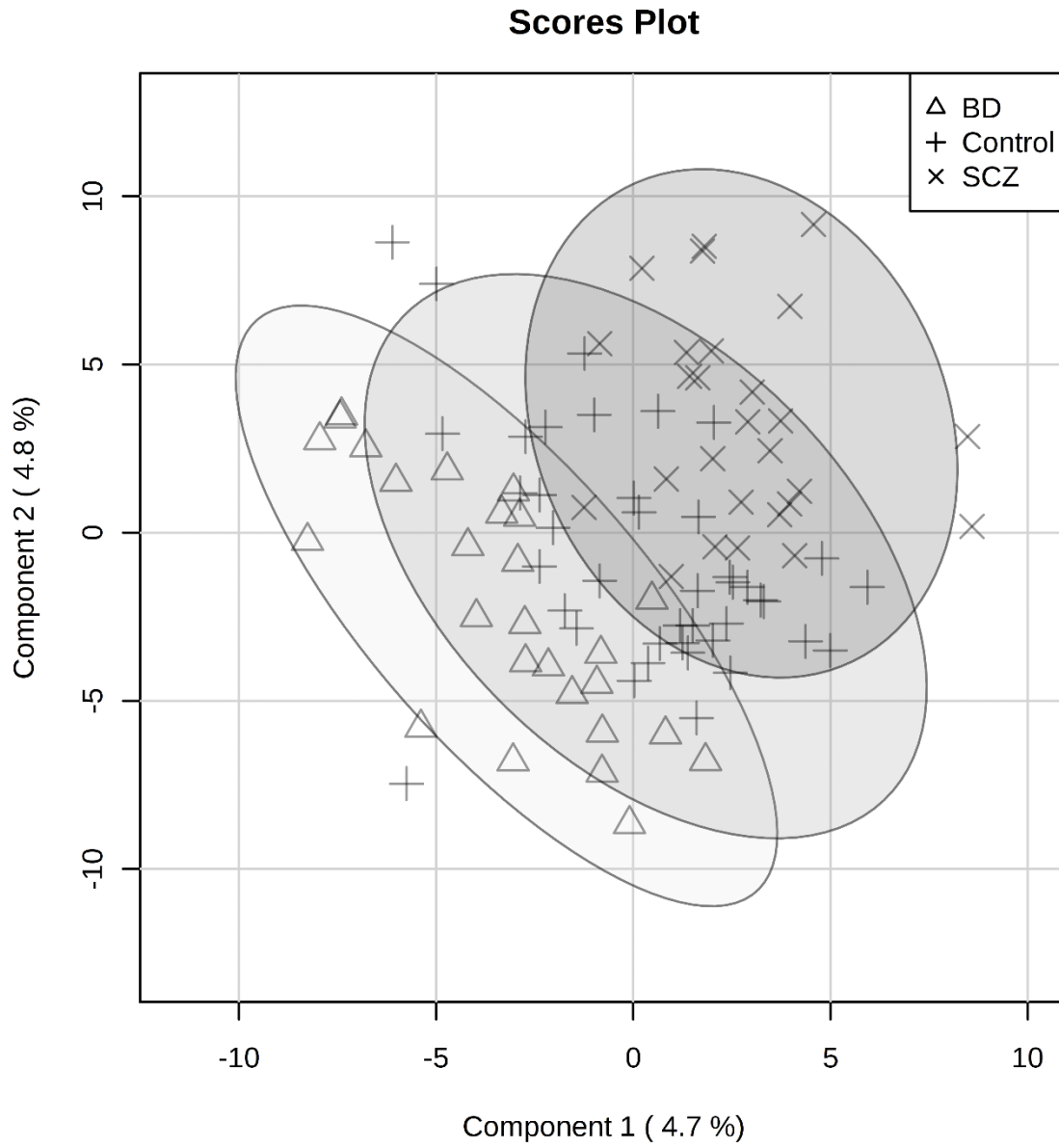

Supplement: Supplementary Figure 1 — PCA (A) and PLS-DA (B) scores plot of SCZ (gray), BD (light gray) and HC (white) samples. [file Image_1.pdf]

2A)

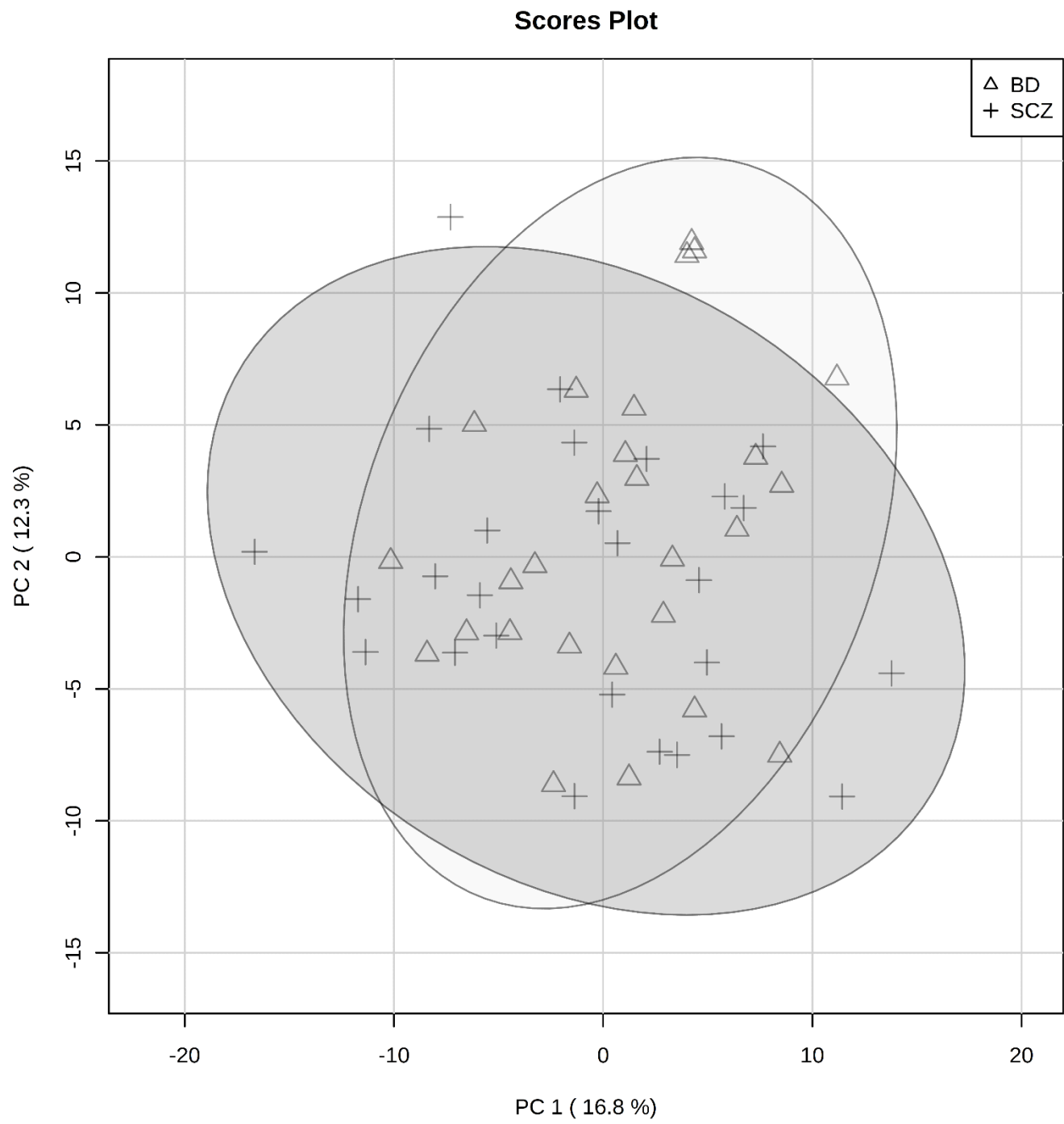

2B)

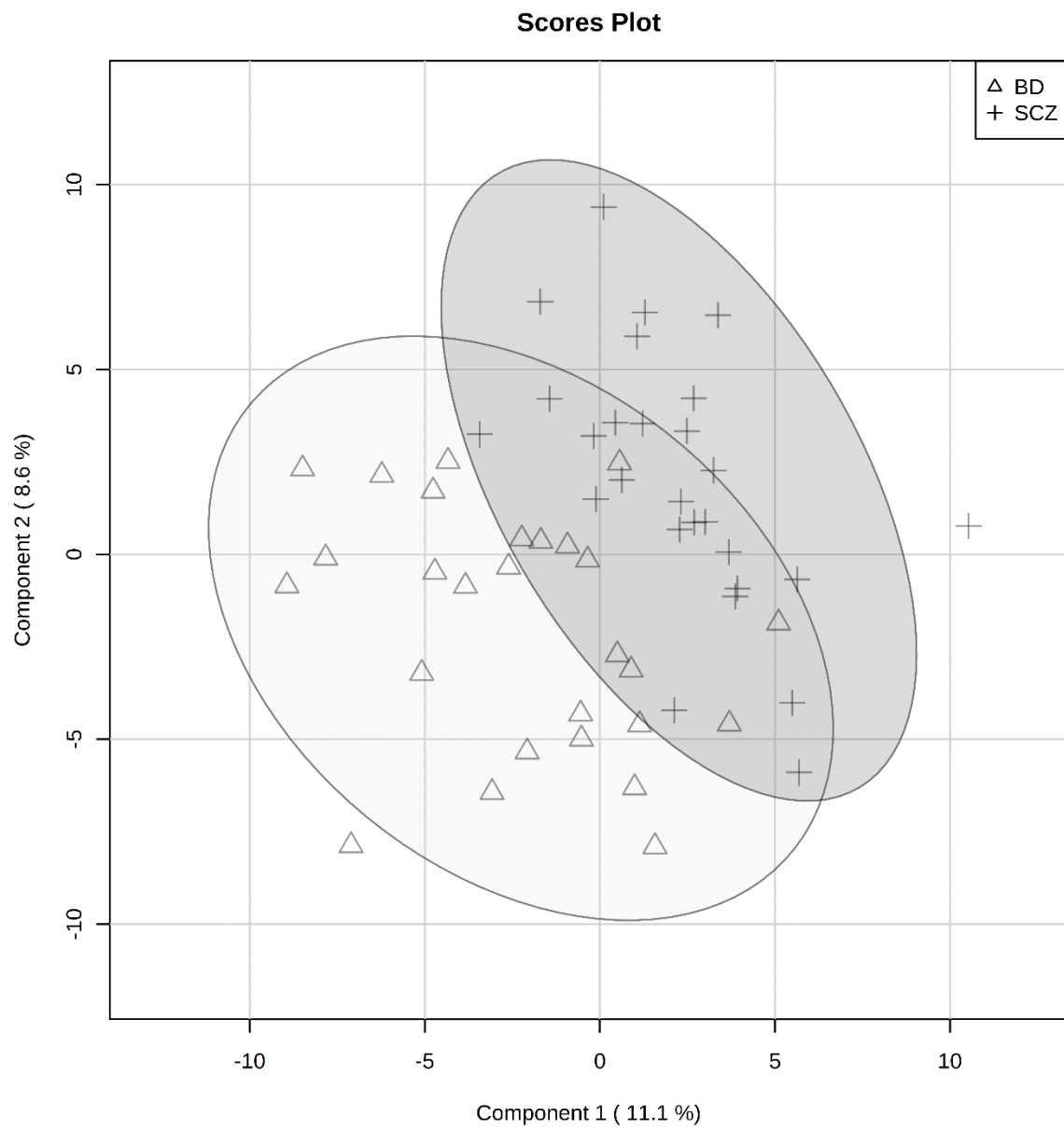

Supplement: Supplementary Figure 2 — PCA (A) and PLS-DA (B) scores plot of SCZ (gray) and BD (light gray) samples. [file Image_2.pdf]
